# Both p62/SQSTM1-HDAC6-dependent autophagy and the aggresome pathway mediate CDK1 degradation in human breast cancer

**DOI:** 10.1038/s41598-017-10506-8

**Published:** 2017-08-30

**Authors:** María Galindo-Moreno, Servando Giráldez, Carmen Sáez, Miguel Á. Japón, Maria Tortolero, Francisco Romero

**Affiliations:** 1 Departamento de Microbiología, Facultad de Biología, Universidad de Sevilla, Seville, E-41012 Spain; 2 Instituto de Biomedicina de Sevilla (IBIS), Hospital Universitario Virgen del Rocío/CSIC/Universidad de Sevilla, Seville, E-41013 Spain; 3 Departamento de Anatomía Patológica, Hospital Universitario Virgen del Rocío, Seville, E-41013 Spain

## Abstract

Cyclin-dependent kinase 1 (CDK1) is the central mammalian regulator of cell proliferation and a promising therapeutic target for breast cancer. In fact, CDK1 inhibition downregulates survival and induces apoptosis. Due to its essential role, CDK1 expression and activity are strictly controlled at various levels. We previously described that CDK1 stability is also regulated and that SCF(βTrCP) ubiquitinates CDK1, which is degraded via the lysosomal pathway. In addition, in breast tumors from patients, we found a negative correlation between CDK1 accumulation and βTrCP levels, and a positive correlation with the degree of tumor malignancy. This prompted us to study the molecular mechanism involved in CDK1 clearance. In this report, we determine that both chemotherapeutic agents and proteolytic stress induce CDK1 degradation in human breast cancer MCF7 cells through p62/HDAC6-mediated selective autophagy. On the one hand, CDK1 binds to p62/SQSTM1-LC3 and, on the other hand, it interacts with HDAC6. Both complexes are dependent on the presence of an intact βTrCP-binding motif on CDK1. Furthermore, we also show that CDK1 is recruited to aggresomes in response to proteasome inhibition for an extended period. We propose CDK1 clearance as a potential predictive biomarker of antitumor treatment efficacy.

## Introduction

Cyclin-dependent kinase 1, CDK1, is a conserved serine/threonine kinase whose activity controls cell cycle progression. In fact, in most mammalian cells, it is the only CDK that is essential for driving the cell cycle^1^. Moreover, CDK1 is highly expressed in tumor tissues^2, 3^, although in some cases, the loss of expression of cytoplasmic CDK1 is associated with a poor prognosis for non-small cell lung cancer patients^4^. Given its important role for the cell, CDK1 activity is tightly regulated: CDK1 has to be associated with a cyclin to recognize and phosphorylate its substrates; CDK1 itself is regulated by phosphorylation, which causes changes in its subcellular localization; and CDK1/cyclin complexes are regulated by their direct binding to CDK inhibitors (for a review, see Malumbres & Barbacid^5^). Furthermore, although its transcription and translation oscillate in a cell cycle-specific manner^6^, CDK1 protein levels remain relatively constant during the cell cycle, indicating that CDK1 stability is an another important level of CDK1 activity regulation.

We previously identified CDK1 as a novel βTrCP-binding protein: CDK1 is ubiquitinated by SCF(βTrCP) and degraded via the lysosomal pathway^7^. Furthermore, the lysosome was also involved in the degradation of CDK1 after proteasome blockage, but in this case the responsible ubiquitin ligase remains unknown. In addition, we noticed that CDK1 accumulation in tumors of different sites showed a negative correlation with βTrCP levels and a positive correlation with the degree of tumor malignancy, demonstrating the importance of controlling the stability of this protein in tumors^7^.

Macroautophagy, referred to hereafter as autophagy, is a conserved eukaryotic catabolic pathway that degrades a wide variety of substrates (or cargo) via the lysosomal pathway^8^. Selective autophagy is characterized by the formation of a double membrane autophagosome that envelops specific cargo proteins in a process dependent on receptor proteins. p62/SQSTM1 protein, hereafter referred to as p62, was the first selective autophagy adaptor protein discovered in mammals. Other cargo receptors have since been described, including NBR1, NDP52, and OPTN^9^. Cargo receptors bind the cargo to an ATG8-family protein through their LC3-interacting region (LIR). LC3B is the most studied ATG8-family protein, which is synthesized as pro-LC3 and immediately processed into a cytosolic form, LC3-I. Conjugation of phosphatidylethanolamine to the carboxy-terminus of LC3-I defines the LC3-II form that is tightly associated with the autophagosomal membrane. Autophagosomes fuse to the lysosome to form an autolysosome with an internal acidic and hydrolytic environment that helps to degrade the content^10^.

Histone deacetylase 6 (HDAC6) plays a double role in the autophagosomal/lysosomal pathway. Firstly, it controls the fusion of autophagosomes to lysosomes by promoting F-actin remodeling in a cortactin-dependent manner^11^. Secondly, upon proteasome inhibition, HDAC6 is recruited and relocates to polyubiquitin-positive aggresomes. In fact, HDAC6 binds both polyubiquitinated proteins and dynein motors for transport to aggresomes^12^. HDAC6 is also involved in protein recruitment in an ubiquitin-independent manner^13^.

Despite the critical role of CDK1 in mammalian cell cycle progression and its importance in tumor formation, the mechanism by which CDK1 is degraded via the lysosomal pathway is unknown. Herein, we demonstrate that both p62/HDAC6-dependent autophagy and the aggresome pathway mediate CDK1 clearance in human breast cancer.

## Results

### Chemotherapeutic agents and proteolytic stress induce CDK1 degradation in human breast cancer MCF7 cells

**Figure 1:**
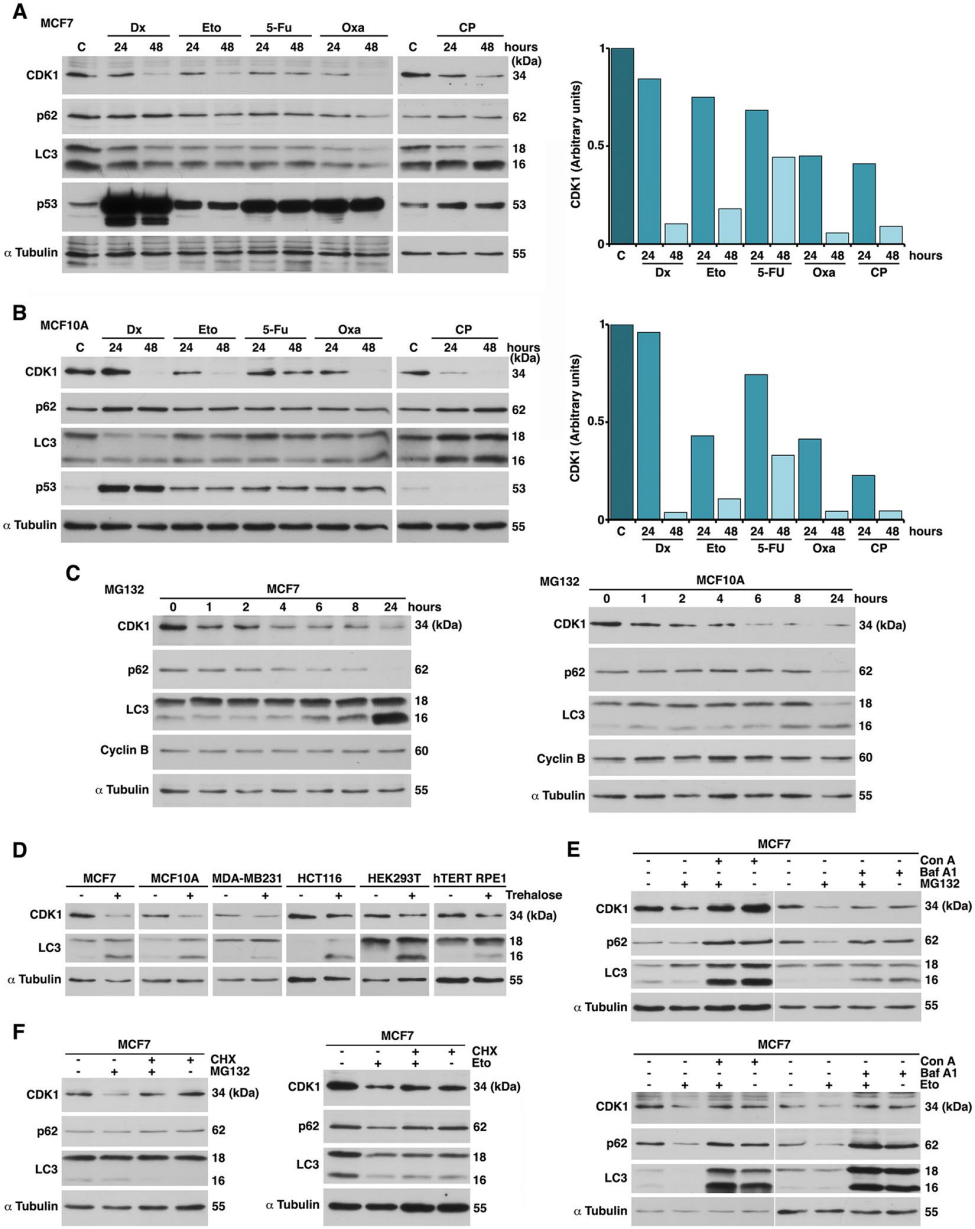
DNA-damaging agents and proteasome inhibitors induce CDK1 degradation via the lysosomal pathway. Exponentially growing MCF7 (**A**) and MCF10A (**B**) cells were treated with the indicated agents and extracts were transferred and immunoblotted with the indicated antibodies. (**C**): extract without any treatment. Dx: doxorubicin; Eto: etoposide; 5-Fu: 5-fluorouracil; Oxa: oxaliplatin; CP: cyclophosphamide. Graphs show quantification of CDK1 protein levels using ImageJ software. (**C**) MCF7 and MCF10A cells were treated with or without the proteasome inhibitor MG132 at the indicated time points. Western blots were performed on lysates using the indicated antibodies. (**D**) MCF7, MCF10A, MDA-MB231, HCT116, HEK293T, and hTERT RPE1 cells were treated or not with trehalose for 24 hours, and samples analyzed by immunoblots. (**E**) MCF7 cells were pretreated with concanamycin A (Con A) or bafilomycin A1 (Baf A1) for 30 min, and then treated with MG132 (4 hours) or etoposide (Eto, 24 hours). Lysates were analyzed by Western blot. (**F**) MCF7 cells were pretreated with cycloheximide (CHX, 30 min) and treated as in (**E**).

To elucidate the molecular mechanisms responsible for CDK1 degradation via the lysosomal pathway, we used MCF7 cells as a model of human breast cancer. Our previous results demonstrated that treatment with the chemotherapeutic agent doxorubicin induced CDK1 degradation via the lysosomal pathway in a cell line-dependent manner. In addition, the proteasome block also provoked the lysosome-mediated CDK1 degradation in all cell lines tested^7^. In this study, we analyzed the effect of both treatments on CDK1 protein levels from MCF7 cells. We used a series of DNA-damaging cancer chemotherapeutic drugs: cyclophosphamide, an alkylating agent; doxorubicin, an anthracycline; etoposide, a topoisomerase II inhibitor; 5-fluorouracil, a nucleotide analog; and oxaliplatin, a platinum-based agent^14–17^. All of the DNA-damaging agents caused a diminution of CDK1 levels (Fig. 1A). Similar results were obtained in MCF10A cells, a non-tumorigenic cell line derived from human fibrocystic mammary tissue that has the characteristics of normal breast epithelium (Fig. 1B).

Proteolytic stress caused by impairment of the ubiquitin-proteasome system (UPS) by MG132, a proteasome inhibiting agent, also triggered a reduction of CDK1 levels (Fig. 1C). To confirm that autophagy activation is involved in CDK1 degradation in the MCF7 cell line, cells were cultivated in a medium containing trehalose, a glucose-glucose disaccharide linked by an α-α-1–1-glycoside bond, which has the ability to mitigate protein aggregate accumulation by stimulating cellular autophagy in several cell types^18, 19^. Trehalose treatment increased the autophagic flux (ratio of LC3-II to LC3-I^20^) both in MCF7 and in other cell lines, and in all cases there was a reduction of CDK1 levels (Fig. 1D).

We checked whether preincubation of cells with concanamycin A (Con A) or bafilomycin A1 (Baf A1), which act by increasing the intralysosomal pH, and consequently, inactivating pH-dependent lysosomal proteases^20^, would prevent CDK1 degradation caused by both MG132 and etoposide. Preincubation (30 min) with Con A or Baf A1 avoided CDK1 degradation induced by 4 hours of MG132 or 24 hours of etoposide treatment (Fig. 1E). Cycloheximide (CHX) pretreatment of MCF7 cells, which blocks translation as well as autophagy^21^, also prevented the degradation of CDK1 (Fig. 1F). Taken together, these data show that chemotherapeutic agents and proteolytic stress induce CDK1 degradation via the lysosomal pathway in MCF7 cells.

### CDK1 degradation involves p62/HDAC6-mediated selective autophagy

**Figure 2:**
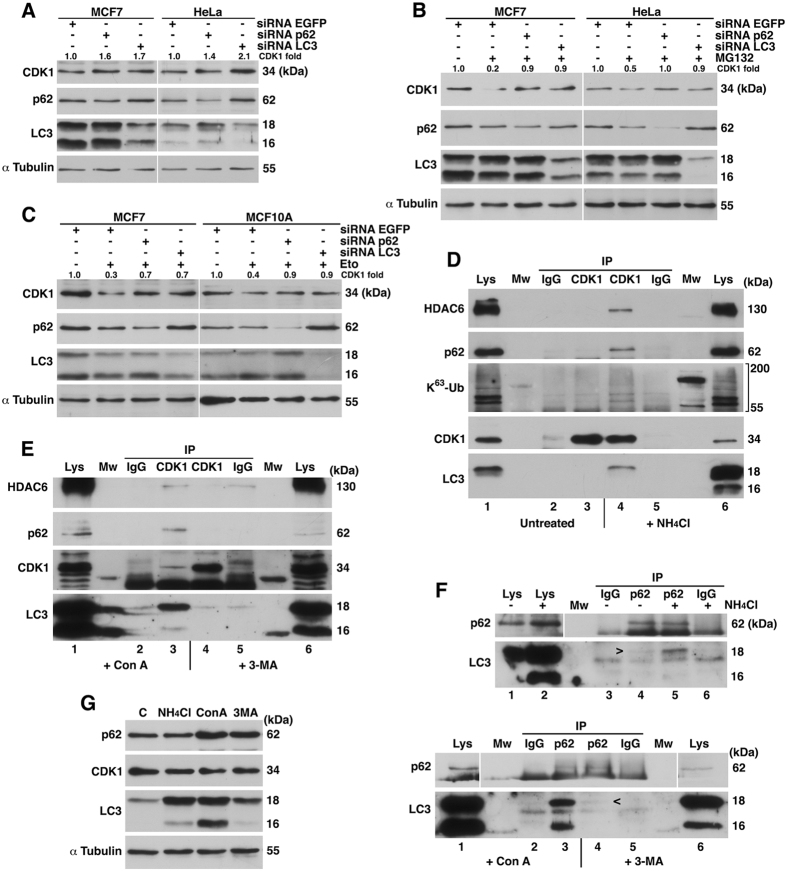
CDK1 degradation involves p62/HDAC6-mediated selective autophagy. (**A**) MCF7 and HeLa cells were interfered with the indicated siRNAs, and extracts were blotted with the designated antibodies. (**B**) MCF7 and HeLa cells interfered with the indicated siRNAs were treated with MG132 4 hours before harvesting, and lysates were subjected to Western blot. (**C**) MCF7 and MCF10A cells were interfered and treated with etoposide (Eto) 24 hours before harvesting. Extracts were blotted with the indicated antibodies. (**D**) NP40 extracts from MCF7 cells treated or not with ammonium chloride (NH_4_Cl) for 24 hours were used to immunoprecipitate CDK1, and the obtained complexes were analyzed by immunoblotting. IP IgG: immunoprecipitation with normal mouse serum used as a control; Lys: Lysate from MCF7 cells untreated or treated with NH_4_Cl; K^63^-Ub: immunoblot using an anti-K^63^-ubiquitin polyclonal antibody that recognizes Lys63-linkage specific ubiquitination; Mw: molecular weight. Lanes are numbered from 1 to 6. (**E**) Co-immunoprecipitation experiments similar to (**D**) but using MCF7 cells treated with concanamycin A (Con A; 1 mg of extract protein) or 3-methyladenine (3-MA; 5 mg of extract protein) for 24 hours. (**F**) Extracts from exponentially growing MCF7 cells treated with NH_4_Cl, Con A or 3-MA for 24 hours were used to immunoprecipitate p62, or normal rabbit serum as a control (IgG). Immunocomplexes were analyzed by Western-blot. Lys: Lysates from MCF7 cells in the different conditions; Mw: molecular weight. Lanes are numbered from 1 to 6. Vertical white lines indicate that bands were taken from different exposures of the same anti-p62 blot. Full-length blots are given in Supplementary Fig. S2. (**G**) Comparison of p62, CDK1, and LC3 levels in MCF7 cells after the indicated treatments for 24 hours. C: Lysate from MCF7 untreated cells; NH_4_Cl: ammonium chloride; Con A: concanamycin A; 3-MA: 3-methyladenine. Quantitative fold change in CDK1 was determined relative to loading control.

To investigate the mechanism by which CDK1 is degraded by autophagosomal/lysosomal pathway, we depleted LC3, an essential molecule for regulating autophagosome biogenesis, and p62, a key protein in the autophagic clearance of polyubiquitinated proteins^9^. We found that the downregulation of both p62 and LC3 increased CDK1 levels in MCF7 cells (Fig. 2A). Similar results were obtained in HeLa cells. Next, we analyzed the CDK1 degradation induced by proteasome block (Fig. 2B) or etoposide treatment (Fig. 2C) in cells depleted of p62 or LC3, and we found that the degradation of CDK1 was avoided in both cases. Comparable results were obtained by silencing *atg5*, which is essential for autophagosome formation (Supplementary Fig. S1). These results indicate that i) CDK1 undergoes basal degradation via autophagy/lysosome, which is increased after etoposide or MG132 treatment, and ii) p62 and LC3 are involved in this turnover.

In order to confirm these observations, we evaluated the binding of CDK1 to p62 and/or LC3 by immunoprecipitation experiments. Autophagic flux was blocked with ammonium chloride, which raises the pH and inhibits lysosomal proteases^22^. CDK1 associated with p62 and LC3 in an immunocomplex, but no binding was observed in untreated cells (Fig. 2D). p62 displays a preference for binding K63-polyubiquitinated substrates^23^. To check whether, in our experimental conditions, CDK1 had K63-linked polyubiquitin chains conjugated to it, we blotted the immunoprecipitation experiments with an anti-ubiquitin K63 specific antibody. In Fig. 2D (third panel), a smear of K63-ubiquitinated proteins is observed only in the CDK1 immunoprecipitates from ammonium chloride-treated MCF7 cells.

HDAC6 also plays an important role in autophagy, recognizing polyubiquitinated proteins and controlling the fusion process of autophagosomes to the lysosome^11^. Thus, we wanted to know whether HDAC6 was associated to CDK1 24 hours after lysosome inhibition. The first panel of Fig. 2D shows that CDK1 and HDAC6 form a complex *in vivo*. Therefore, after ammonium chloride treatment, CDK1 interacts with both p62/LC3 and HDAC6.

The formation of both complexes was verified by incubating MCF7 cells with Con A (Fig. 2E, left). However, when we inhibited autophagy by blocking autophagosome formation with 3-methyladenine (3-MA), we were not able to detect CDK1 immunocomplexes (Fig. 2E, right), even when the amount of immunoprecipitated CDK1 was greatly increased (compare CDK1 levels from lane 3 *vs*. 4 in Fig. 2E). These data indicate that CDK1 degradation is mediated by the autophagosomal/lysosomal pathway, but complexes can only be visualized when autophagic flux is blocked.

To confirm these results, we analyzed the p62/LC3 complex in our conditions. It is known that the intracellular level of p62 is controlled by constitutive autophagic degradation^24^. p62 was associated with LC3 in all experimental scenarios, although a higher coimmunoprecipitation was detected when lysosomal degradation was blocked (Fig. 2F). Comparing the levels of CDK1 and p62 to those of LC3 in the different extracts (Fig. 2G), we can deduce that under unstressed conditions, only a small proportion of CDK1 and p62 is degraded via the lysosomal pathway, whereas LC3 is clearly the most affected protein. However, the inactivation of lysosomal enzymes allowed for the enrichment of CDK1/p62/LC3 complexes to such an extent that they were detected in the coimmunoprecipitation assays. Accordingly, we conclude that p62/LC3 binds to CDK1 in physiological conditions, mediating its degradation via the lysosomal pathway.

**Figure 3:**
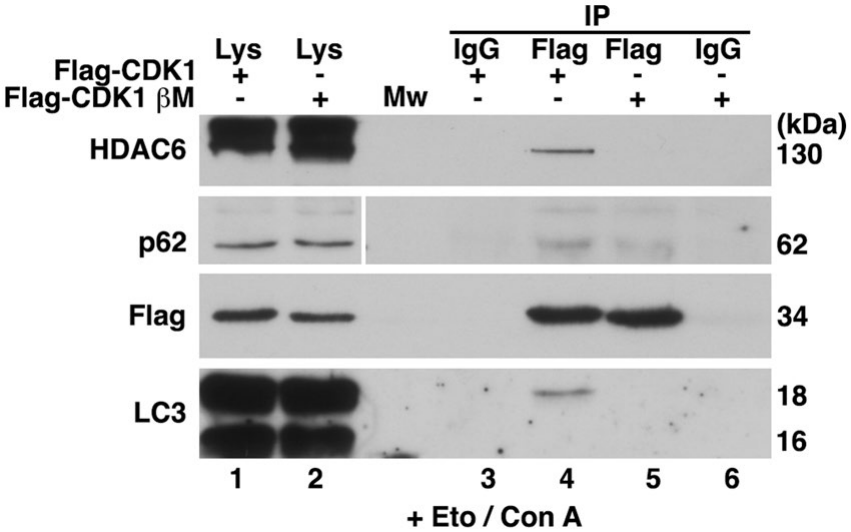
DNA damage induces the association of CDK1 with p62/LC3 and HDAC6 in a βTrCP-dependent manner. HEK293T cells were transfected with pFlagCMV2-CDK1 or pFlagCMV2-CDK1 βM and treated with etoposide (Eto) and concanamycin A (Con A) 24 hours before harvesting. Extracts were used to immunoprecipitate Flag-tagged proteins. Normal mouse serum (IgG) was used as a control. Complexes were analyzed by immunoblotting. Lys: Extracts from HEK293T transfected cells; Mw: molecular weight. Lanes are numbered from 1 to 6. Vertical white line indicates that bands were taken from different exposures of the same anti-p62 blot. Full-length blots are given in Supplementary Fig. S2.

We previously demonstrated that SCF(βTrCP) is the ubiquitin ligase responsible for CDK1 ubiquitination and degradation after DNA damage^7^. To corroborate that the formation of CDK1/p62/LC3 and CDK1/HDAC6 complexes following DNA damage is dependent on the presence of an intact βTrCP-binding motif on CDK1, we used the CDK1 mutant CDK1βM^7^, which cannot associate with βTrCP and thus cannot be ubiquitinated by SCF(βTrCP). HEK293T cells were transfected with pFlagCMV2 CDK1 or pFlagCMV2 CDK1βM and, after 24 hours, incubated with etoposide (and Con A to avoid CDK1 degradation) for 24 hours. We then analyzed the formation of CDK1 complexes. After Eto + Con A treatment, Flag-CDK1 interacts with p62/LC3 and HDAC6, but the Flag-CDK1βM mutant does not (Fig. 3). This confers a determining role to SCF(βTrCP) on the association of CDK1 with p62/LC3 and HDAC6 after DNA damage. Together, these experiments demonstrate that CDK1 is degraded by p62/HDAC6-dependent autophagy, and this degradation increases after DNA damage in an SCF(βTrCP)-dependent manner.

### CDK1 degradation mediated by both proteasome inhibition and DNA damage takes place in the autophagosome/lysosome-enriched cellular fraction

**Figure 4:**
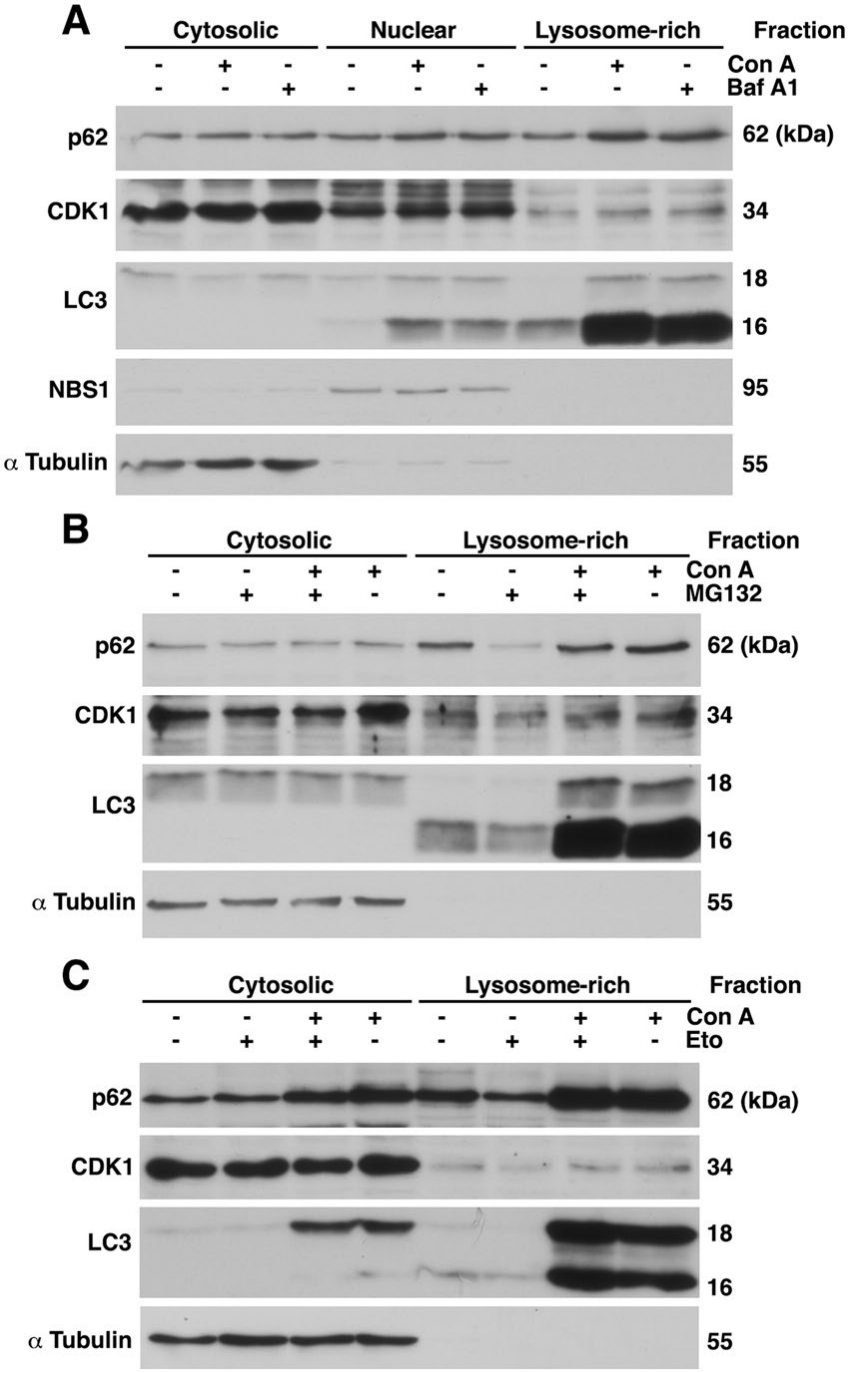
Location of CDK1 in the autophagosome/lysosome-enriched cellular fraction. (**A**) Subcellular fractions were prepared from MCF7 cells treated or not with concanamycin A (Con A) or bafilomycin A1 (Baf A1) for 4.5 hours, as described in Materials and Methods. Equal amounts of protein were analyzed by SDS-PAGE and immunoblot. The fractions were tested for purity against α Tubulin (cytosolic fraction), NBS1 (nuclear fraction), and LC3 (lysosome rich fraction). (**B**, **C**) MCF7 cells were pretreated with Con A for 30 min and then treated with MG132 (4 hours) (**B**) or etoposide (Eto, 24 hours) (**C**). Subcellular fractions prepared as in (**A**) were analyzed by Western blot.

To assess whether CDK1 is engulfed in autophagosomes for its further degradation, we first analyzed if CDK1 is present in the so-called lysosome-enriched cellular fraction^22^, which also includes small vesicles enclosed by a biological membrane. MCF7 cells were maintained in normal growth medium, or Con A or Baf A1 treatment for 4 hours, and subcellular fractions prepared and analyzed by Western blot (Fig. 4A). We found that CDK1 was partially located in the lysosomal fraction under control conditions, and that the amount of CDK1 in this fraction increased when lysosomal proteases were inhibited, corroborating a basal CDK1 degradation through the lysosome. Similar results were obtained with p62 (Fig. 4A). The enrichment in each fraction was verified by analyzing the presence of α Tubulin in the cytosol, NSB1 in the nucleus, and the preferential distribution of LC3-II in the lysosomal fraction.

Next, we examined the effect of proteasome inhibition (Fig. 4B) or etoposide treatment (Fig. 4C) on the presence of CDK1 in the lysosome-enriched cellular fraction. We found a decrease of CDK1 in both cases, which was avoided by inhibiting the lysosomal enzymes with Con A. We observed similar results when p62 degradation was studied. These data indicate that CDK1 is engulfed into autophagosomes after proteolytic stress or DNA damage to be degraded via the lysosomal pathway.

### CDK1 is recruited to aggresomes in response to proteasome inhibition but not to DNA damage

**Figure 5:**
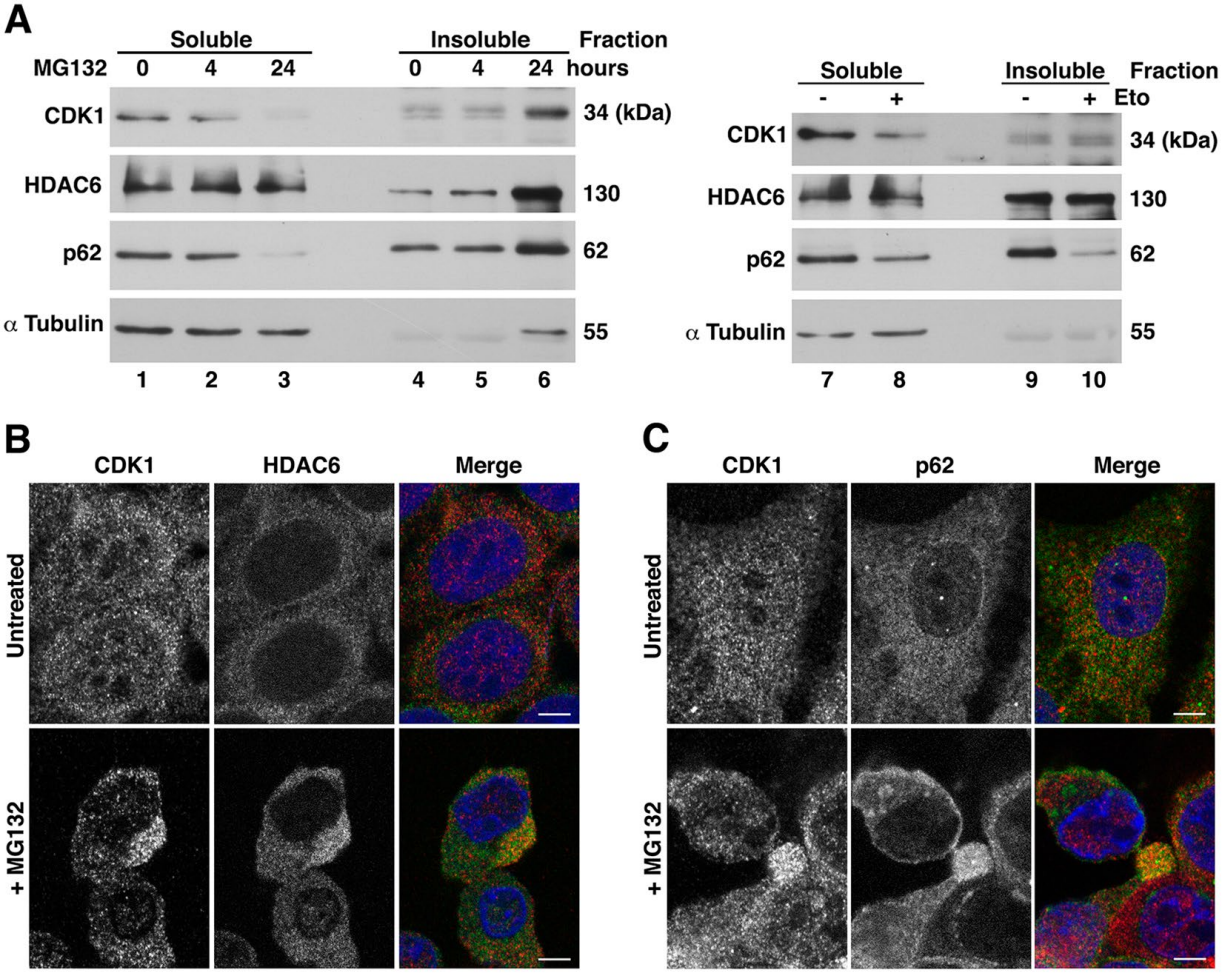
CDK1 is recruited to aggresomes in response to proteasome inhibition. (**A**) NP40-soluble and -insoluble fractions from MCF7 cells treated with MG132 for the indicated times or etoposide (Eto) for 24 h were analyzed by immunoblot. Lanes are numbered from 1 to 10. (**B**, **C**) MCF7 cells treated or not with MG132 for 24 hours were analyzed by confocal microscopy. In the merge, CDK1 staining is shown in red and HDAC6 (**B**) or p62 (**C**) in green. Bars represent 5 μm.

To investigate whether the solubility of CDK1 was altered after etoposide or MG132 treatment, cellular lysates were fractionated into NP40-soluble and -insoluble fractions. No change was observed in the solubility of CDK1 in our conditions (4 hours of MG132 or 24 hours of etoposide) (Fig. 5A, lanes 4 *vs*. 5, and 9 *vs*. 10) indicating that activation of the autophagosomal/lysosomal pathway was enough to induce CDK1 clearance.

It was previously shown that inhibition of the UPS for an extended period can induce aggresome formation coordinated by HDAC6^12^. To investigate whether CDK1 could also form insoluble aggregates, we inhibited the proteasomes of MCF7 cells by 24 hours of MG132 treatment. The level of CDK1 dramatically increased in the NP40-insoluble fraction (Fig. 5A, lanes 4 *vs*. 6). Similarly, p62 and HDAC6 accumulated in the insoluble fraction after 24 hours of proteasome block. These results suggest that when autophagy is overloaded due to proteasome inhibition, CDK1 is located in the detergent*-*insoluble fraction, coinciding with the aggresome formation.

Next, we used microscopy to confirm that CDK1 associated with aggresomes in MCF7 cells after MG132 treatment for 24 hours. We found that CDK1 concentrated close to a perinuclear structure whose morphology and localization resemble that of an aggresome (Fig. 5B). Supporting these data, confocal microscopy examination revealed that endogenous CDK1 and HDAC6 colocalized in this structure after proteasome inhibition, whereas both proteins were diffused across untreated cells. Furthermore, CDK1 also colocalized with p62 (Fig. 5C), a protein known to be required for aggresome formation in metabolically stressed conditions^24^. Our results strongly suggest that when the cell cannot degrade CDK1 by the autophagosomal/lysosomal pathway, CDK1 accumulates in the aggresome to be subsequently degraded by autophagy.

## Discussion

CDK1 is an essential kinase for cell cycle progression, being the only CDK whose elimination arrests the cell cycle in mammalian cells^1^. CDK1 interacts with cyclin B to drive the G2/M transition but also with other cyclins to regulate G1 progression and the G1/S transition. CDK1 is overexpressed in many cancers, such as oral squamous cell carcinoma, esophageal adenocarcinoma, gastric cancer, liver cancer, colorectal cancer, ovarian cancer, and breast cancer^25^. Specifically, breast cancer is one of the most common malignancies in women, and was used in this report as a model system to study CDK1 degradation via the lysosomal pathway.

Herein, we show for the first time the molecular mechanism by which CDK1 is degraded via the lysosomal pathway. We found that chemotherapeutic agents or proteolytic stress in human breast cancer cells induced CDK1 degradation mediated by p62/LC3 and HDAC6. The fact that both p62 and LC3 depletion increases CDK1 levels or prevents its degradation suggests that CDK1/p62/LC3 proteins could be forming a complex, where CDK1 would bind to p62, and p62 to LC3. This would have to be the case because if there were a direct binding of CDK1 to LC3, p62 downregulation would not increase CDK1 levels^26, 27^. In addition, although CDK1 presents a putative LIR domain for the direct association to LC3 (residues 4 to 7), we presume that it is not functional, since the CDK1 mutant that cannot bind to βTrCP contains the unchanged LIR domain and did not associate with LC3 after DNA damage.

Moreover, when the autophagosomal/lysosomal pathway is overloaded (which occurs, for example, when the proteasome is blocked for a long period of time), CDK1 is accumulated into aggresomes to be later degraded. These results are supported by the fact that CDK1 acts in a very similar way to p62. We observed that short-term proteolytic stress (4 hours) induced CDK1 and p62 degradation, an effect which was especially visible in lysosome-rich extracts, and was avoided with Con A or Baf A1 treatment. However, after 24 hours of proteasome block, but not after DNA damage, CDK1, p62 and HDAC6 were located in the aggresome, probably to avoid the accumulation of cytotoxic protein aggregates^28^.

The CDK1 degradation observed in both MCF7 and MCF10A cell lines when the cells were treated with DNA-damaging agents agrees with the efficacy of chemotherapy treatments for breast tumors. Depending on the type of breast cancer, several different types of drugs are used. Among the most common drugs used before (neoadjuvant chemotherapy) or after (adjuvant chemotherapy) surgery, or for advanced breast cancer, are doxorubicin, 5-fluorouracil, cyclophosphamide, and platinum agents^14, 29–31^. Therefore, it is not surprising that these agents target CDK1, most likely among others. In fact, when DNA damage does not reduce CDK1 levels, cells are more resistant to cell death, and thus the treatment would become ineffective^7^.

We also noticed that after treatment with any DNA-damaging agents, p62, in addition to CDK1, is degraded via the lysosomal pathway, and its degradation is also reversed with lysosomal proteases inhibitors. These data corroborate previously published results showing that p62 is degraded after ionizing radiation, but not in autophagy-deficient *ATG3* knockout cells^32^. The authors propose that p62 contributes to avoid or reduce DNA repair by down-regulating RNF168 ubiquitin ligase, responsible for histone H2A ubiquitination. This is necessary for the recruitment of the downstream regulators of the DNA double-strand break response pathway. Therefore, autophagy-defective tumor cells accumulate p62 and are associated with genomic instability^33^. Based on the results obtained in this study and those previously published, we can conclude that in cells with a functional autophagy system, the reduction of p62 and CDK1 caused by DNA damage not only allows for DNA repair but probably arrests the cell cycle in order to give time to repair the damages.

CDK1 is considered as an important therapeutic target because its inhibition downregulates survival and induces apoptosis. That is the strategy that has been followed with the estrogen, progesterone, and HER2 receptor-negative triple-negative breast cancer, which is characterized by its biological aggressiveness and poor prognosis^34^. These tumors exhibit elevated c-MYC expression, and the authors used *CDK1* siRNA delivered by cationic lipid assisted nanoparticles to induce decreased cell viability and apoptosis only in c-MYC overexpressed triple-negative breast cancers and not in normal mammary epithelial cells. Thus, reducing CDK1 levels could be a measure of treatment effectiveness. Of no less importance, the combined effects of the proteasome inhibitor bortezomib with HDAC inhibitors should also be further investigated since the accumulation of cytotoxic protein aggregates that cannot form aggresomes may induce cell death.

In summary, the findings presented in this report provide important novel mechanistic insights into CDK1 degradation via the lysosomal pathway. Our results also underline the importance of tumor subtypes and cellular context in terms of therapies based on DNA-damaging agents. We propose that the levels of CDK1 clearance could potentially be used as a predictive biomarker for the efficacy of breast cancer chemotherapy.

## Methods

### Cell cultures and lysis

MCF7, MDA-MB231, HCT116, HeLa and HEK293T (from ATCC, Manassas, VA, USA) were grown in Dulbecco’s modified Eagle’s medium (DMEM) (BioWest, Nuaillé, France) as described^35^. MCF10A cells were grown in DMEM/F12 medium (BioWest) with L-glutamine and HEPES, supplemented with 5% (v/v) horse serum, 100 μg/ml streptomycin and 100 U/ml penicillin from Gibco (Thermo Fisher Scientific, Waltham, MA, USA), and 20 ng/ml epidermal growth factor, 0.5 μg/ml hydrocortisone, 100 ng/ml cholera enterotoxin and 10 μg/ml insulin from Sigma-Aldrich (St. Louis, MO, USA). hTERT RPE1 cells were grown in DMEM/F12 medium (BioWest) with L-glutamine and HEPES, supplemented with 5% (v/v) fetal bovine serum, 100 μg/ml streptomycin and 100 U/ml penicillin from Gibco. NP40 extracts were prepared at 4 °C in 150 mM NaCl, 10 mM Tris-HCl (pH 7.5), 1% Nonidet P-40 (NP40), 10% glycerol, 1 mM PMSF (phenylmethylsulfonyl fluoride), 1 μg/ml aprotinin, 1 μg/ml pepstatin, 1 μg/ml leupeptin and 10 μg/ml chymostatin for 20 min. Extracts were centrifuged at 20,000 *g* for 20 min and supernatants frozen in liquid nitrogen and stored at −80 °C. Protein concentration was determined using the Bradford assay (Bio-Rad, Hercules, CA, USA).

### Drugs

For some experiments, cells were treated with doxorubicin (Dx, 1 μM, Sigma-Aldrich), etoposide (Eto, 10 μM, Sigma-Aldrich), 5-fluorouracil (5-FU, 10 μM, Sigma-Aldrich), oxaliplatin (Oxa, 10 μM, European Pharmacopoeia Reference Standards, Strasbourg, France), cyclophosphamide (CP, 10 mM, Sigma-Aldrich), MG132 (20 μM, Santa Cruz Biotechnology, Dallas, TX, USA), trehalose (100 mM, Sigma-Aldrich), concanamycin A (Con A, 50 nM, Sigma-Aldrich), bafilomycin A1 (Baf A1, 200 nM, Santa Cruz Biotechnology), cycloheximide (CHX, 50 μg/ml, Sigma-Aldrich), ammonium chloride (40 mM, Sigma-Aldrich) or 3-methyladenine (3-MA, 10 mM, Merck, Darmstadt, Germany) at the indicated times.

### Electrophoresis, Western blot analysis and antibodies

Equal amounts (20–30 μg) of proteins were subjected to 10–12% SDS-polyacrylamide gel electrophoresis (SDS-PAGE) and gels were electroblotted onto nitrocellulose membranes and probed with the following antibodies: anti-CDK1 mouse (1:1,000) and anti-HDAC6 rabbit (1:1,000) monoclonal, and anti-K63-linkage specific polyubiquitin rabbit (1:500) polyclonal antibodies (Cell Signaling Technology, Danvers, MA, USA); anti-p62 (1:1,000), anti-LC3 (1:400) and anti-NBS1 (1:1,500) rabbit polyclonal antibodies (Novus Biologicals, Littleton, CO, USA); anti-Flag (1:5,000) and anti-α Tubulin (1:20,000) mouse monoclonal, and anti-ATG5 rabbit (1:1,000) polyclonal antibodies (Sigma-Aldrich); and anti-p53 (1:500) and anti-cyclin B (1:1,000) mouse monoclonal antibodies (Santa Cruz Biotechnology). Peroxidase-coupled donkey anti-rabbit IgG (1:10,000) and sheep anti-mouse IgG (1:10,000) were obtained from GE Healthcare (Little Chalfont, UK). Immunoreactive bands were visualized using the Enhanced Chemiluminescence Western blotting system (ECL, GE Healthcare). Quantification of protein levels was carried out using ImageJ software (Image Processing and Analysis in Java; National Institutes of Health, Bethesda, MD, USA; http://imagej.nih.gov/).

### Small interfering RNA (siRNA) assays

Cells were interfered with p62-, LC3-^36, 37^ or ATG5-siRNA (Qiagen, Hilden, Germany) using the Oligofectamine method (Invitrogen, Carlsbad, CA, USA) to suppress the expression of endogenous genes. EGFP-siRNA^38^ was used as a non-specific control. Cells were harvested 48 hours post-transfection and reduction of protein levels confirmed by Western blotting.

### Co-immunoprecipitation experiments

NP40 extracts (1–2 mg) were incubated with normal mouse or rabbit sera for 30 minutes and subsequently with protein G or A-sepharose beads (GE Healthcare), respectively, for 1 hour at 4 °C. After centrifugation, beads were discarded and supernatants incubated for 2 hours with anti-CDK1 (Cell Signaling Technology), anti-Flag (Sigma-Aldrich) or normal mouse (Santa Cruz Biotechnology) monoclonal antibodies or serum, or anti-p62 (Novus Biologicals) polyclonal antibody or normal rabbit (Santa Cruz Biotechnology) serum, followed by protein G or A-sepharose beads for 1 hour. Beads were washed and bound proteins were solubilized by the addition of SDS-sample buffer heated at 95 °C for 5 minutes.

### Transient transfections

pFlagCMV2-CDK1 or pFlagCMV2-CDK1 βM^7^ were transiently transfected using a lipid transfection reagent (Xfect, Clontech, Mountain View, CA, USA); 24 hours post-transfection, cells were incubated with etoposide and concanamycin A for 24 hours, harvested, and lysed.

### Subcellular fractionation

Fractions were prepared according to Li and coworkers^22^, with minor modifications. Briefly, MCF7 cells were resuspended in hypotonic buffer (40 mM KCl, 5 mM MgCl_2_, 2 mM EGTA, 10 mM HEPES, pH 7.5) for 30 min on ice. Later, cells were homogenized by shearing through a 28.5-gauge needle 30 times. After centrifugation at 1,000 *g* for 10 min, the resulting pellet was the nuclear fraction. The supernatant was then centrifuged at 12,000 *g* for 10 min. This second supernatant was the cytosolic fraction, while the pellet, enriched for the lysosome, was further washed in an isotonic buffer (150 mM NaCl, 5 mM MgCl_2_, 2 mM EGTA, 10 mM HEPES pH 7.5) and dissolved in a lysis buffer (1% Triton X-100, 150 mM NaCl, 50 mM Tris-HCl pH 7.5) for further analysis. Nuclear and cytosolic fractions were also lysed with 1% Triton X-100.

### NP40-soluble and -insoluble fractionation

Following drug treatment, MCF7 cells were lysed on ice in NP40 lysis buffer containing 420 mM NaCl. After centrifugation at 15,000 *g* for 30 min at 4 °C, NP40-soluble fractions were collected. The pellets were washed four times with the same lysis buffer and further solubilized in the same volume of SDS sample buffer (62.5 mM Tris-HCl pH 6.8, 2% SDS, 10% glycerol, 5% β-mercaptoethanol, 0.05% bromophenol blue) and sonicated. NP40-insoluble fractions were collected by centrifugation at 15,000 *g* for 30 min at 4 °C. Equal amounts of protein from soluble fractions, and the corresponding volume of insoluble fractions, were blotted as described above.

### Confocal microscopy analysis

Cells were grown on coverslips, fixed in 4% paraformaldehyde for 10 min at room temperature and permeabilized with 0.1% Triton X-100. Then, cells were incubated with primary antibodies for 1 hour at room temperature, washed with 0.1% PBS-Tween, and incubated with the appropriate fluorescent secondary antibody for 45 min. Nuclei were counterstained with DAPI (100 ng/ml, Sigma-Aldrich) after secondary antibody labeling. Staining was analyzed using a Zeiss LSM 7 DUO confocal microscope, and images were acquired under a 63x oil-immersion objective at a definition of 1,024 × 1,024 pixels with the pinhole diameter adjusted to 0.9 μm. All images were acquired using the same laser parameters and image magnification. Images were processed using Photoshop (Adobe Systems Incorporated, San José, CA, USA).

## Electronic supplementary material

Supplementary figures
