# Supplementary material for: Both p62/SQSTM1-HDAC6-dependent autophagy and the aggresome pathway mediate CDK1 degradation in human breast cancer
Source: Sci Rep. 2017 Aug 30;7:10078. doi: 10.1038/s41598-017-10506-8 (PMC5577189; doi:10.1038/s41598-017-10506-8)

Both p62/SQSTM1-HDAC6-dependent autophagy and the aggresome pathway mediate CDK1 degradation in human breast cancer

María Galindo-Moreno[1], Servando Giráldez[1], Carmen Sáez[2,3], Miguel Á. Japón[2,3], Maria Tortolero[1], and Francisco Romero[1,*]

[1] Departamento de Microbiología, Facultad de Biología, Universidad de Sevilla, Seville, E-41012, Spain
[2] Instituto de Biomedicina de Sevilla (IBIS), Hospital Universitario Virgen del Rocío/CSIC/Universidad de Sevilla, Seville, E-41013, Spain
[3] Departamento de Anatomía Patológica, Hospital Universitario Virgen del Rocío, Seville, E-41013, Spain

SUPPLEMENTARY INFORMATION

Supplementary Figures and Legends

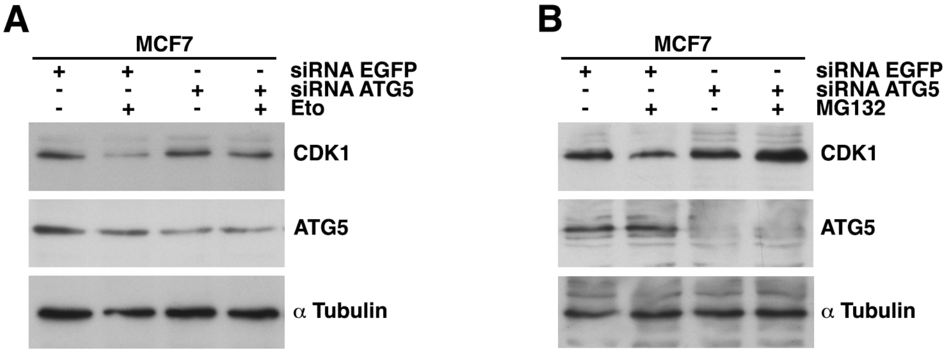

Supplementary Figure S1. Silencing of *atg5* avoids CDK1 degradation induced by etoposide or MG132. (A) MCF7 cells were interfered with the indicated siRNAs and treated with etoposide (Eto) 24 hours before harvesting. Lysates were subjected to Western blot. (B) MCF7 cells interfered as in (A) were treated with MG132 4 hours before harvesting. Extracts were blotted with the indicated antibodies.

Supplementary Figure S2. Full-length from Figure 2F and Figure 3.

Figure 2F (anti-p62)

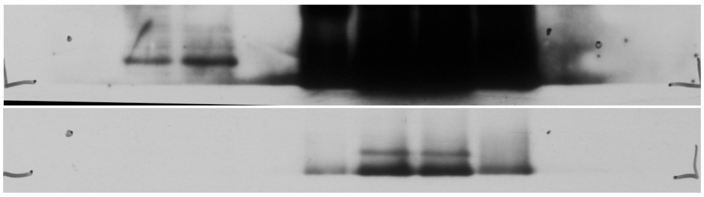

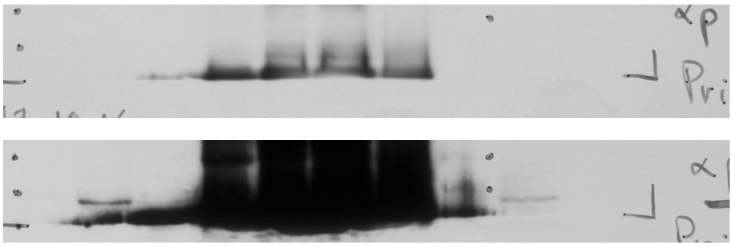

Figure 3 (anti-p62)

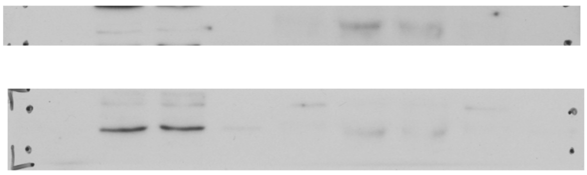

Supplement: Supplementary file 1 — Supplementary figures [file 41598_2017_10506_MOESM1_ESM.pdf]
